# With a little help from T cells

**DOI:** 10.7554/eLife.106854

**Published:** 2025-04-30

**Authors:** Troy Burtchett, Neal Hammer

**Affiliations:** 1 Department of Microbiology, Genetics, and Immunology, Michigan State University East Lansing United States

**Keywords:** MRSA, microbiota, gastrointestinal colonization, sex hormone, Th17, Mouse

## Abstract

Specific host factors, such as immune cell activity, sex hormones and microbiota composition, influence the ability of *Staphylococcus aureus* bacteria to colonize the gut of mice.

**Related research article** Lejeune A, Zhou C, Ercelen D, Putzel G, Yao X, Guy AR, Pawline M, Podkowik M, Pironti A, Torres VJ, Shopsin B, Cadwell K. 2024. Sex-dependent gastrointestinal colonization resistance to MRSA is microbiota and Th17 dependent. *eLife* **13**:RP101606. doi: 10.7554/eLife.101606.

Antibiotic-resistant bacteria such as methicillin-resistant *Staphylococcus aureus* (or MRSA for short) are a leading cause of morbidity and mortality worldwide and present a large burden for hospitals and other healthcare facilities (Murray et al., 2022). Although around a third of the population carries *S. aureus* – including MRSA – in the nose or on the skin without any symptoms, if these bacteria enter the body, they can cause serious, often life-threatening infections (Sakr et al., 2018; Castleman et al., 2018).

Recent studies have shown that the gut can also serve as a reservoir of MRSA (Piewngam and Otto, 2024), and around 20% of the healthy population carries the bacteria in the gut. This asymptomatic MRSA colonization ha been associated with increased risks of bloodstream infections and is also linked to increased transmission through inanimate objects in both the community and hospital settings (Squier et al., 2002). However, little is known about the strategies MRSA uses to colonize the gut and why some people are more susceptible to this colonization than others. Now, in eLife, Ken Cadwell and colleagues – including Alannah Lejeune as first author – report new insights into the factors that influence gut colonization of MRSA (Lejeune et al., 2024).

The researchers (who are based at New York University, the University of Pennsylvania and St. Jude Children’s Research Hospital) studied two different populations of laboratory mice bred at New York University (NYU mice) and Jackson Laboratories (JAX mice). Female mice bred at NYU were able to clear MRSA from their gut, while both male and female JAX mice and NYU male mice were persistently colonized. These results suggest that the microbiota (the community of microbes residing within the gut) may affect colonization rates of MRSA, which is in keeping with findings from previous studies focusing on other pathogens (Caballero-Flores et al., 2023).

Analyzing the microbiota of the different mice revealed that female NYU mice harbored a distinct composition of microbes compared to JAX mice (Figure 1). Moreover, when female JAX mice were housed with female NYU mice to allow transfer of microbiota between the mice, they were able to clear MRSA. However, male and female NYU mice were found to have similar microbiota, indicating that other factors can also influence MRSA colonization in the gut. This is similar to findings in epidemiological studies in humans showing that men are more frequently colonized with MRSA, demonstrating that the mouse model can be used to represent human colonization patterns accurately (Humphreys et al., 2015).

**Figure 1.**
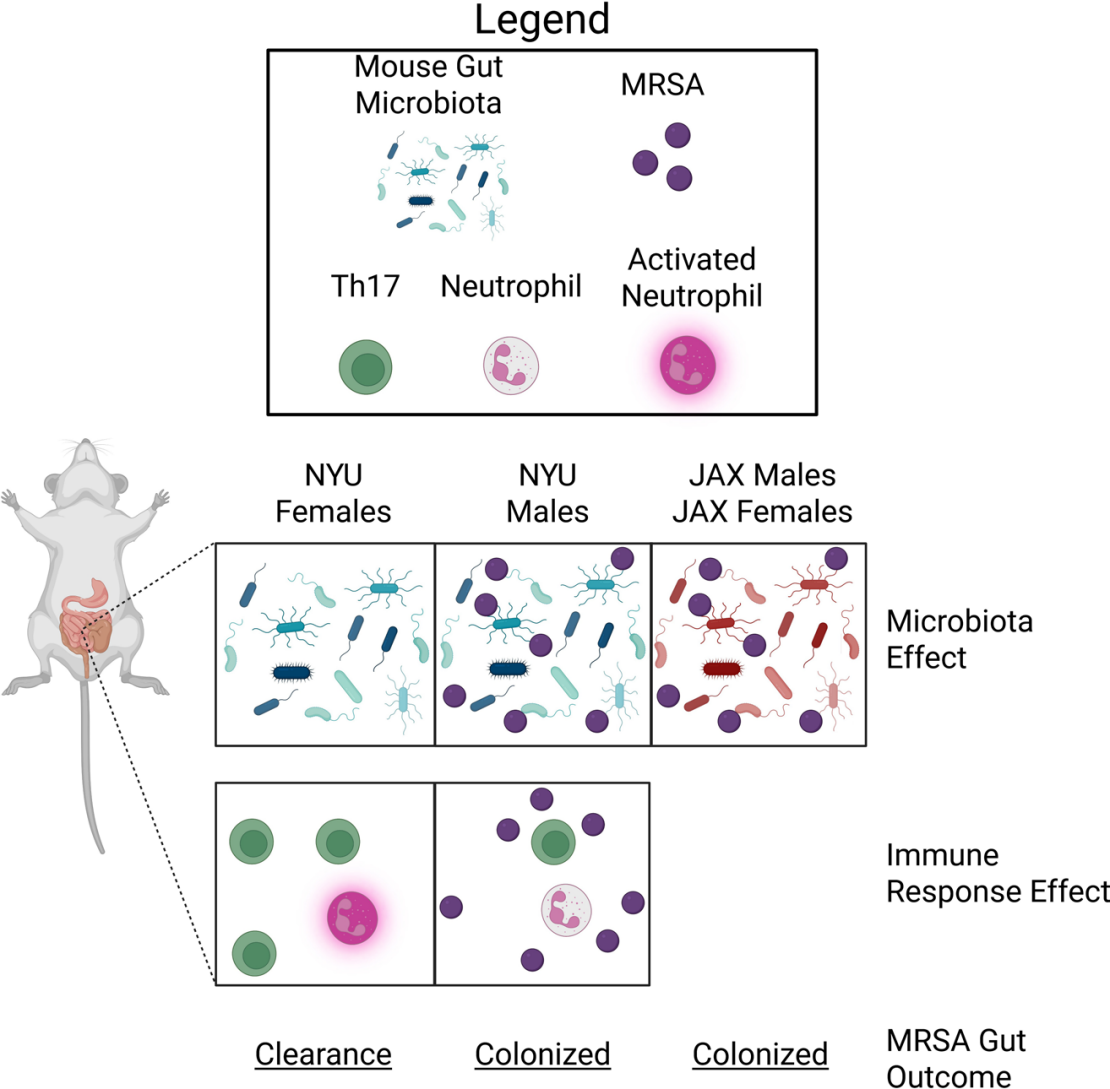
MRSA colonization in the gut of mice is microbiota- and sex-dependent. The gut microbiota of female and male mice bred at New York University (NYU) or Jackson Laboratories (JAX) influences the ability of MRSA bacteria to colonize the gut. The gut microbiota of NYU female mice (left panel) differs from NYU male mice (middle panel) and both male and female JAX mice (right panel). This difference provided NYU females with unique protection against MRSA colonization (microbiota effect). The immune response of NYU females after they were infected with MRSA resulted in increased counts of T-helper 17 (Th17) cells (green) and activated neutrophils (pink), which promoted clearance of MRSA (immune response effect; bottom panel). Th17 cell proliferation and neutrophil activation were not observed in NYU male mice, resulting in persistent colonization with MRSA.

Next, Lejeune et al. wanted to identify sex-dependent factors that lead to the differences observed between female and male mice. The researchers analyzed gene expression patterns that respond to MRSA colonization in the gut of male and female NYU mice, including genes associated with the activity and migration of immune cells. Male and female mice had distinct gene expression patterns. Most notably, females upregulated genes associated with T cell and neutrophil activity, cells involved with long-term immunity and rapid immune response, respectively (Sun et al., 2023).

The T cell response was unusual given the short MRSA exposure, but Lejeune et al. confirmed the importance of T cells in clearing MRSA from the gut of female mice using genetically modified mice and cell depletion assays. In particular, a specific type of T-helper cell known as the Th17 cell, which is important for activating neutrophils, was crucial for clearing MRSA (Figure 1). This supports previous findings indicating that neutrophils play a critical role in controlling MRSA colonization and infection throughout the body (Rigby and DeLeo, 2012).

To determine the mechanism of the sex-dependent immune activation in the gut, Lejeune et al. focused on two features of females that are distinct from males: the additional X chromosome and female sex hormones. Transplanting female XX immune cells into male counterparts did not eliminate MRSA from the gut of male mice, but removing ovaries, the source of female sex hormones, reversed MRSA clearance from the gut in females. Consistent with this, female mice lacking the estrogen receptor had increased levels of MRSA in their gut. Lastly, an elegant transgenic mouse experiment that allowed hormonal and chromosomal distinctions between males and females to be further decoupled provided additional evidence that female hormones promote MRSA clearance. Overall, the work demonstrates that the microbiota, an enhanced Th17 cell response and female sex hormones play pertinent roles in the clearance of MRSA from the gut.

Important considerations for future research include determining whether specific microbes within the microbiota stimulate Th17 cells in an estrogen-dependent manner to activate neutrophils that clear MRSA. Another outstanding question is whether other pathogens can be cleared from the gut via a similar mechanism. New knowledge gained using this model can be leveraged to develop new decolonization strategies to reduce the risk of invasive infections. Perhaps most importantly, this work serves as a poignant example that investigating sex as a biological variable in the context of host-pathogen interactions can lead to important discoveries.
